# Peptide Mimetic Platform for Activity Profiling of Mycobacterial l,d‑Transpeptidases

**DOI:** 10.1021/acs.biochem.6c00126

**Published:** 2026-05-22

**Authors:** Karl L. Ocius, Rachel E. Sanborn, Ananya Naick, Leighanne A. Brammer Basta, Marcos M. Pires

**Affiliations:** † Department of Chemistry, 2358 University of Virginia, Charlottesville, Virginia 22904, United States; ‡ Chemistry Department, 32722 United States Naval Academy, Annapolis, Maryland 21402, United States; § Department of Microbiology, Immunology, and Cancer, University of Virginia, Charlottesville, Virginia 22904, United States

## Abstract

Antimicrobial resistance poses major therapeutic challenges, particularly for multidrug-resistant mycobacterial infections caused by *Mycobacterium tuberculosis* (*Mtb*) and nontuberculous mycobacteria (NTM). l,d-Transpeptidases (Ldts) are attractive drug targets due to their essential role in peptidoglycan cell wall cross-linking, yet existing assays suffer from low throughput and limited sensitivity. We report a versatile, bead-based platform for high-throughput analysis of Ldt activity and inhibitor discovery. We incubated peptidoglycan stem peptides, either naturally harvested or synthetically immobilized on abiotic surfaces, with Ldts and a fluorescent acyl acceptor to quantitatively monitor cross-linking. After optimizing assay parameters, we profiled six *Mycobacterium smegmatis* Ldt paralogs, including the first characterization of a class 6 Ldt with chemically defined substrate sequences. Utilizing a series of acyl acceptors, we demonstrated modifications within the acyl acceptor that are tolerated by mycobacterial Ldts. Screening of β-lactam antibiotics revealed potent inhibition by (carba)­penems, while cephalosporins, monobactams, and penams showed negligible activity. The assay achieved excellent performance metrics and was successfully adapted to ELISA and 96-well formats, providing a powerful tool for discovering Ldt-targeted therapeutics against tuberculosis and related infections.

## Graphical abstract

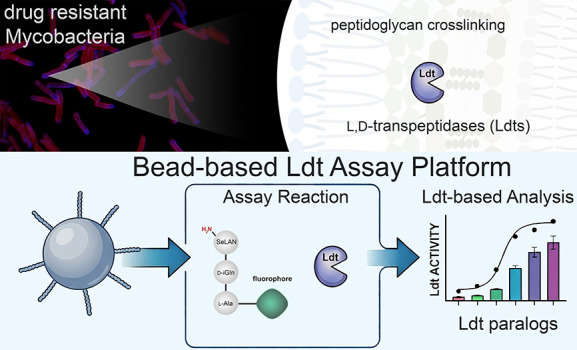

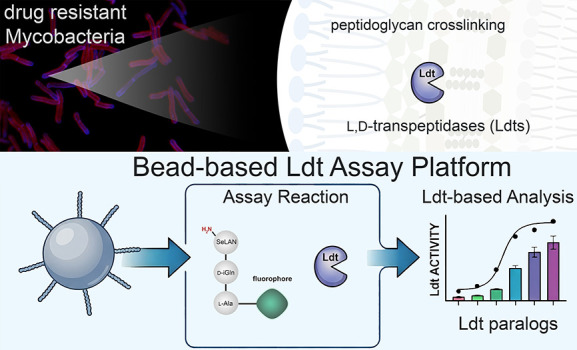

## Introduction

Tuberculosis (TB), caused by the bacterium *Mycobacterium tuberculosis* (*Mtb*), remains one of the world’s deadliest infectious diseases, claiming 1.23 million lives in 2024 alone.^1^ The disease burden is unevenly distributed across patient populations, with TB representing the leading cause of mortality among people living with HIV.^2,3^ The emergence of multidrug-resistant (MDR) and extensively drug-resistant (XDR) strains continues to erode therapeutic options,^1,4^ while infections caused by nontuberculous mycobacteria (NTM) are rising worldwide.^5^ Together, these trends underscore an urgent need for novel antimycobacterial agents. Yet, antibiotic development has stagnated, and the broader antimicrobial resistance crisis is projected to cause 10 million deaths annually by 2050.^6^ Addressing this challenge requires not only new drug candidates but also innovative tools to identify and validate them.

The bacterial cell wall, specifically its peptidoglycan (PG) layer, has long served as a validated therapeutic target. This mesh-like structure provides mechanical strength and defines cellular shape, making it essential for bacterial survival.^7,8^ In mycobacteria, PG architecture has glycan strands composed of alternating *N*-acetylglucosamine and *N*-acetylmuramic acid that are cross-linked by peptide bridges, creating a complex and inherently heterogeneous macromolecular network.^9^ The muramic acid residues are often *N*-glycolylated, a modification that has recently been shown to be more potently sensed by the pathogen-associated molecular pattern (PAMP) receptor NOD2.^10,11^ A prominent feature of PG is the high degree of cross-linking observed between neighboring peptide strands. Two enzyme families catalyze these cross-links: the d,d-transpeptidases (penicillin-binding proteins, PBPs) and the l,d-transpeptidases (Ldts) (Figure 1a).^9^ Unlike PBPs, which utilize a serine-based catalytic mechanism to form 4→3 peptide cross-links, Ldts employ a cysteine-based mechanism to generate 3→3 cross-links between adjacent tetrameric peptide stems generated by carboxypeptidases (Figure 1b). The first step in Ldt-catalyzed cross-linking involves acylation of the active site cysteine by the carbonyl of the acyl donor substrate (donor strand), forming a covalent acyl-enzyme intermediate, and releasing the terminal group d-alanine (d-Ala). At this intermediate stage, there is a thioester intermediate between the Ldt and the truncated donor strand. Next, the ε-amino group of *meso*-diaminopimelic acid (*m*-DAP) from an adjacent acceptor peptide strand attacks this activated acyl intermediate, generating the cross-link and regenerating free Ldt enzyme.^12^

**Figure 1:**
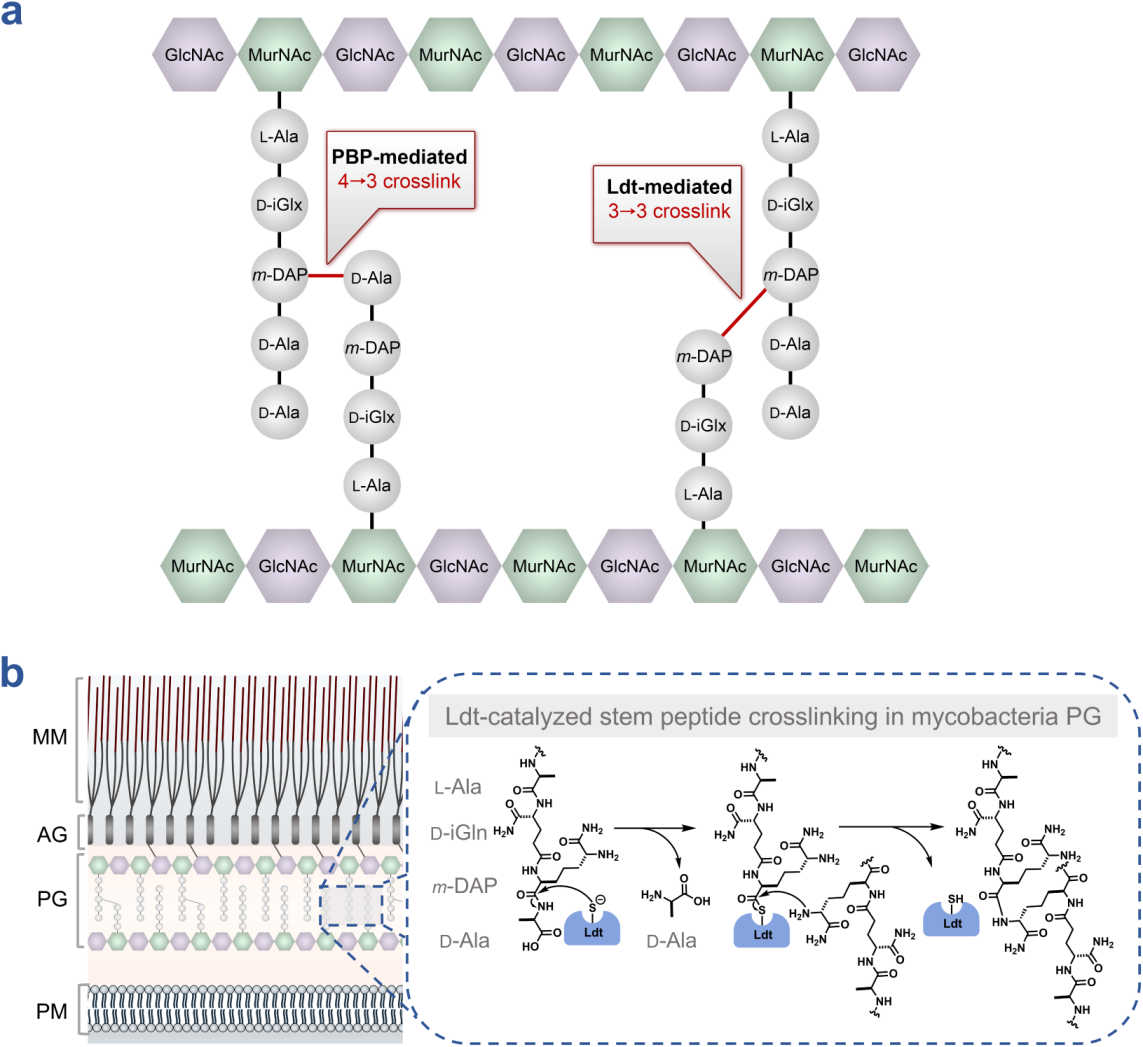
Composition and stem peptide cross-linking within the cell wall of mycobacteria. (**a**) PG consists of glycan strands composed of alternating *N*-acetylglucosamine (GlcNAc) and *N*-acetylmuramic acid (MurNAc), to which a stem peptide is covalently attached. Adjacent stem peptides are cross-linked to form a macromolecular network. Cross-linking is catalyzed by two enzyme families: d,d-transpeptidases (penicillin-binding proteins, PBPs), which generate 4→3 cross-links, and l,d-transpeptidases (Ldts), which generate 3→3 cross-links. (**b**) The mycobacterial cell envelope comprises the mycomembrane (MM), arabinogalactan (AG), peptidoglycan (PG), and plasma membrane (PM). Ldts catalyze stem peptide cross-linking within the periplasm via a two-step mechanism. The catalytic cysteine first performs a nucleophilic attack on a donor stem peptide, forming an enzyme–peptide intermediate and releasing the *C*-terminal d-alanine (d-Ala). The ε-amino group of the *meso*-diaminopimelic acid (*m*-DAP) residue of an adjacent acceptor stem peptide then attacks this intermediate, resulting in the formation of a covalent 3→3 cross-link and regeneration of the free enzyme.

While mycobacteria encode both enzyme classes, Ldts predominantly mediate PG cross-linking in these species and are essential for cell wall integrity, making them critical therapeutic targets; however, their unique catalytic mechanism and limited susceptibility to classical β-lactams make them challenging to inhibit. Genetic deletion studies have confirmed that Ldts are essential for mycobacterial survival, with their absence severely compromising cell wall stability and restricting bacterial growth.^13–17^ These phenotypic observations establish Ldts as validated therapeutic targets whose inhibition could effectively expand the arsenal of antimycobacterial agents to combat TB infections. However, Ldts are notoriously difficult to inhibit due to their unique active site architecture, which restricts β-lactam access and often results in unstable or readily reversible covalent adducts.^18–22^ This challenge is compounded by chromosomally encoded β-lactamases in *Mtb* and NTM species that hydrolyze and inactivate many β-lactams before they reach their targets,^23–26^ leaving only carbapenems and penems as effective Ldt inhibitors. While recent clinical success with β-lactam/β-lactamase inhibitor combinations has renewed interest,^27–30^ the systematic discovery of novel Ldt inhibitors remains hampered by significant methodological limitations.

A major obstacle to Ldt-targeted drug discovery has been the difficulty in characterizing enzyme activity, which requires the synthesis of peptidyl substrates containing *m*-DAP, a building block that remains challenging to produce using standard solid-phase peptide synthesis.^31^ Compounding this challenge, existing assays are insufficient to accurately monitor the two-step transpeptidation reaction and characterize the 3→3 cross-linked products formed during catalysis, which are often polymeric and heterogeneous in nature.^14,32–34^ There have been prior efforts guided toward the analysis of broad inhibition of Ldts, which have attempted to circumvent these challenges. Thiol-reactive colorimetric and fluorogenic probes have been used to indirectly monitor inhibition by assessing overall cysteine reactivity; however, these approaches provide little insight into features unique to Ldts.^32,35^ Similarly, d-alanine oxidase (DAAO) has been repurposed to detect the release of d-Ala that accompanies the first step of the transpeptidation reaction.^36^ More recently, a rotor-fluorogenic d-amino acid probe was shown to report on changes in bond rotation upon cross-linking, enabling detection of both Ldt and PBP activity.^37^ Finally, recent studies have revealed the range of detectable transpeptidase targets using fluorescently labeled β-lactams combined with gel-based imaging.^38–43^

Given the possible redundancy of Ldts in many organisms, including mycobacteria, which encode ∼5–6 individual Ldts, it remains to be elucidated how individual transpeptidases (including PBPs) operate within the inherent heterogeneity of PG matrices. Some studies suggest, at least in terms of specific PG labels, that the transpeptidation with analogs of the stem peptide could be specific to a single PBP in *S. aureus* ^44^ and a single Ldt in *E. coli*.^45^ We hypothesized that specific PG strand structures temporally and spatially coordinate Ldt activity. The extent of this structural variation is substantial; a single bacterial strain can display over 250 unique muropeptides.^46^ We, and others, recently demonstrated that amidation of *iso*-glutamic acid with the muropeptide is critical for transpeptidase processing in live cells.^47–50^ Given this vast range of unique PG structures within a single cell, Ldt processing could be modulated, to varying extents, by the muropeptide primary sequence.

Existing assays for evaluating Ldt inhibition and reactivity with covalent modifiers rarely incorporate substrates that reflect the structural complexity of native PG, limiting mechanistic insight into Ldt function. To address this gap, we developed a high-throughput transpeptidation platform that reconstitutes and quantifies both steps of Ldt catalysis. A synthetic acyl-donor peptide is immobilized on solid supports (or native sacculi), and enzyme-mediated incorporation of a fluorescent acyl-acceptor is quantified by flow cytometry. This configuration captures the full catalytic cycle (acylation of the enzyme by the bead-bound donor followed by nucleophilic resolution by the fluorescent acceptor), yielding a cross-linked product whose formation is directly reported by fluorescence levels. Active-site Ldt inhibitors block cross-linking and produce a proportional loss of signal. Applying this system, we delineated transpeptidase activities and acyl acceptor processing across multiple Ldt paralogs and assessed inhibition by a focused β-lactam library. The compatibility of the assay with both flow-cytometric and ELISA-based detection expands its utility, and its robust performance metrics underscore its value for mechanistic studies and screening applications.

## Experimental Section

### Peptidoglycan/Sacculi Isolation of *M. smegmatis*

Methodology for isolation of sacculi is an adaptation from published protocols.^51–54^ *Mycobacterium smegmatis* strains mc^2^ 155, ATCC 14468 were grown in 7H9 media with 0.5% glycerol, 0.05% Tween 80, and 1× ADC enrichment (10× ADC, 5 g bovine serum albumin, 2 g dextrose, 3 mg catalase in 100 mL deionized water, sterilized by filtration through a 0.2 μm filter before use). *M. smegmatis* was inoculated from the glycerol stock by 1 to 1000 dilution into 3 mL fresh 7H9 media with ADC and grown to the stationary phase. Then, cell pellets were resuspended in 200 μL 10 mM NH_4_HCO_3_ with protease inhibitor and bath-sonicated for 30 min. 10 μg/mL DNase and RNase were added to each well directly after sonication, and the tube was placed at 4 °C for 1 h. The cell wall-enriched fractions were collected by centrifugation at 2700 *g* for 10 min. The pellets were then treated with 200 μL PBS containing 2% sodium dodecyl sulfate (SDS) and incubated at 50 °C for 1 h with shaking at 250 rpm, repeated three times. Finally, the suspension was centrifuged, and the resulting pellet was reconstituted in 200 μL PBS with 1% SDS and 0.1 mg/mL proteinase K, incubated at 45 °C for 1 h with shaking at 250 rpm. The tube with the resulting suspension was then heated in boiling water for 1 h and centrifuged at 2700 g for 10 min. The supernatant was discarded, and the 1% SDS boiling step was repeated twice, followed by centrifugation. The pellets were then washed twice with PBS and 4 times with deionized water to give the mycolyl-arabinogalactanpeptidoglycan complex (MAPc). The pellet was resuspended in 200 μL methanol with 0.5%(w/v) KOH and incubated at 37 °C with shaking at 250 rpm for 4 days. The suspension was then centrifuged, and the pellets were washed with methanol twice and diethyl ether twice and air-dried to afford arabinogalactan-peptidoglycan (AGPG). The resulting AGPG was resuspended in 200 μL deionized water. AGPG was digested with 0.05 N H_2_SO_4_ at 37 °C for 5 days with shaking at 250 rpm, centrifuged, and washed 4 times with deionized water to give insoluble peptidoglycan (PG). The sacculi were recovered by centrifugation at 21,000 × *g* for 20 min, washed 5× with 0.1 M Tris pH 8, resuspended in Milli-Q water, and stored at −20 °C for subsequent analysis.

### Expression of Ldt_Mt2_ Proteins and Purification

*Ldt*_*Mt2*_ was cloned into a modified pET28a vector, expressed, and purified as we reported previously.^33,55^ The plasmid was transformed into *E. coli* BL21­(DE3) cells (NEB). Cultures were grown at 37 °C to an OD_600_ of ∼0.5, cooled to 16 °C, induced with 100 μM IPTG (Isopropyl β-d-1-thiogalactopyranoside) , and incubated with shaking for 20 h. Cells were harvested by centrifugation (3500 × *g*, 10 min, 4 °C), stored at −20 °C, and then resuspended in lysis buffer (25 mM Tris-HCl, pH 8.0, 400 mM NaCl, 10% glycerol, 1 mM TCEP, protease inhibitor cocktail). After sonication, lysates were clarified by centrifugation (24,500 × *g*, 30 min, 4 °C) and applied to Ni-NTA resin for 1 h at 4 °C. Bound protein was eluted over a 20–500 mM imidazole gradient, and fractions containing His_6_-Ldt_Mt2_ were pooled. For tag removal, pooled fractions were dialyzed for 48 h at 4 °C against 25 mM Tris-HCl (pH 8.0), 100 mM NaCl, 10% glycerol, and 1 mM TCEP in the presence of TEV protease (1:100 w/w). Dialysis buffer was exchanged three to four times. The digest was reapplied to Ni-NTA resin, and the flow-through containing tag-free Ldt_Mt2_ was collected. Protein purity was verified by SDS-PAGE, concentrations were determined spectrophotometrically, and aliquots were flash-frozen in liquid nitrogen and stored at −80 °C.

### Expression of *M. smegmatis* Ldts

*M. smegmatis* Ldts were cloned into a modified pET28b vector, expressed, and purified as we reported previously.^56^ Briefly, *E. coli* BL21­(DE3) cells harboring Ldt-pET28b constructs were cultured in LB medium at 20 °C with shaking until OD_600_ reached ∼0.5, at which point protein expression was induced with 100 μM IPTG for 14–20 h. Cells were collected by centrifugation, resuspended in buffer (25 mM Tris, pH 8.0, 400 mM NaCl, 10% glycerol), and lysed by sonication. After clarification, lysates were applied to Ni-NTA resin, and bound proteins were eluted with imidazole. Fractions containing Ldt were identified by SDS-PAGE, pooled, and dialyzed overnight against 50 mM Tris (pH 8.0), 100 mM NaCl, and 10% glycerol in the presence of TEV protease (1:100, w/w). Cleaved proteins were separated from the His_6_-tag and TEV protease by a second Ni-NTA step, followed by dialysis into storage buffer (50 mM Tris, pH 8.0, 100 mM NaCl, 10% glycerol, 1 mM TCEP). Proteins were concentrated to ≥1 mg/mL, flash frozen in liquid nitrogen, and stored at −80 °C.

### Ldt_Mt2_ Catalyzed Cross-Linking of **qSeDAPtri** on *M. smegmatis* Sacculi

Sacculi were pipetted into wells of a round-bottom, untreated 96-well plate containing 25 mM Tris pH 8, 2 μM Ldt_Mt2_, and 20 μM **qSeDAPtri** at room temperature for 5 h. Control wells excluded the enzyme. This experiment was performed in triplicate. Following incubation, the enzymatic reaction was quenched using 0.1% trifluoroacetic acid (TFA). The sacculi were then washed thrice by centrifugation at 4000 × *g* for 3 min with PBS containing 0.01% Tween-20. Sacculi fluorescence was assessed by an Attune NxT flow cytometer using the 488 nm laser and the 525/49 bandpass filter.

### Copper-Catalyzed Azide–Alkyne Cycloaddition (CuAAC) of **tetFamk-yne** onto SPHERO Azido Polystyrene Particles

Sphero azido polystyrene beads 3.31 μm 1% w/v (250 μL) were resuspended in 1× PBS in a round-bottom, untreated 96-well plate, then treated with 100 μM **tetFAMK-yne**, 1 mM CuSO_4_, 128 μM THPTA (tris­(3-hydroxypropyltriazolylmethyl)­amine), 1.2 mM l-ascorbic acid. The negative control included all the reagents except for CuSO_4_. The particles were incubated at 37 °C for 2 h. After incubation, they were washed 3× by centrifugation at 4000 × *g* for 3 min with 1× PBS containing 0.01% Tween-20. Using the reported particle concentration (∼1.35 × 10^8^ particles/mL) and azide density (∼1.10 × 10^8^ azides per particle) provided by spherotech, we estimate ∼1.5 × 10^16^ available azide sites (∼25 nmol) per mL of beads. As the alkyne peptide was used in excess (100 μM), and assuming ∼85% conversion, ∼21 nmol of peptide is expected to be immobilized. Based on an estimated loading of ∼21 nmol of peptide per mL of beads, the effective donor concentration under our assay conditions (5 μL beads in 110 μL total volume) is approximately ∼1 μM, noting that this represents the apparent concentration of immobilized substrate. Bead fluorescence was assessed by an Attune NxT flow cytometer using the 488 nm laser and the 525/49 bandpass filter, as described previously

### Copper-Catalyzed Azide–Alkyne Cycloaddition (CuAAC) of **tetAck-yne** onto SPHERO Azido Polystyrene Particles

Sphero azido polystyrene beads 3.31 μm 1% w/v (250 μL) were resuspended in 1× PBS in Eppendorf tubes, then treated with 100 μM purified acyl donor peptide **tetAcK-yne**, 1 mM CuSO_4_, 128 μM THPTA, and 1.2 mM l-ascorbic acid. The particles were incubated at 37 °C for 2 h. After incubation, they were washed 3× by centrifugation at 4000 × *g* for 3 min with 1× PBS containing 0.01% Tween-20. They were resuspended to a 0.5% w/v suspension in 1× PBS.

### Determination of the Optimal Time Needed for Cross-Linking on Beads

In a round-bottom, untreated 96-well plate, 5 μL of beads bearing 100 μM of **tetAcK-yne** were incubated with 25 mM Tris pH 8, 2 μM Ldt_Mt2_, and 20 μM **qSeDAPtri** at room temperature for 1, 5, 10, 15, 20, 25, 30, 45, 60, 90, and 120 min. The beads were thoroughly mixed by pipetting prior to incubating them with shaking at room temperature for the indicated time. Each condition was run in triplicate. The addition of 0.1% TFA was used to stop enzymatic catalysis at each time point. The beads were subsequently washed thrice by centrifugation at 4000 × *g* for 3 min with PBS containing 0.01% Tween-20. Bead fluorescence was assessed by an Attune NxT flow cytometer using the 488 nm laser and the 525/49 bandpass filter, as described previously.

### Determination of the Optimal Concentration of **tetAck-yne**

Sphero azido polystyrene beads 3.31 μm 0.5% w/v (Spherotech, 50 μL) were washed in PBS, and varying concentrations of purified acyl donor peptide **tetAcK-yne** (50, 100, 500, and 1000 μM) were reacted with the azido beads in PBS containing 1 mM CuSO_4_, 128 μM THPTA, and 1.2 mM l-ascorbic acid. The particles were incubated at 37 °C for 2 h. After incubation, they were washed thrice with 1× PBS and resuspended to a 1% w/v suspension. Then, in a round-bottom untreated 96-well plate (VWR cat. 82050–622), 5 μL of beads bearing the appropriate concentration of **tetAcK-yne** were incubated with 25 mM Tris pH 8, 2 μM Ldt_Mt2_, and 20 μM **qSeDAPtri**. The beads were thoroughly mixed by pipetting prior to incubating them with shaking at room temperature for 1 h. Each of the conditions was run in triplicate. Negative controls were azido beads free of the acyl donor peptide. The addition of 0.1% TFA stopped enzymatic catalysis after the incubation period. The beads were subsequently washed three times by centrifugation at 4000 × *g* for 3 min with PBS containing 0.01% Tween-20. Bead fluorescence was assessed by an Attune NxT flow cytometer with the 488 nm laser and the 525/49 bandpass filter, as described previously.

### Determination of the Optimal Concentration of **qSeDAPtri**

In a round-bottom, untreated 96-well plate, 5 μL of beads bearing 100 μM of **tetAcK-yne** were incubated with 25 mM Tris pH 8, 2 μM Ldt_Mt2_, and varying concentrations of **qSeDAPtri**, respectively 0.1, 0.5, 1, 5, 10, and 20 μM. The beads were thoroughly mixed by pipetting prior to incubating with shaking at room temperature for 1 h. The addition of 0.1% TFA stopped enzymatic catalysis after the incubation period. Each condition tested here was done in triplicate. Negative control wells excluded the addition of the enzyme. The beads were subsequently washed thrice by centrifugation at 4000 × *g* for 3 min with PBS containing 0.01% Tween-20. Bead fluorescence was assessed by an Attune NxT flow cytometer using the 488 nm laser and the 525/49 bandpass filter as described previously.

### Determination of Optimal Enzyme Concentration

In a round-bottom, untreated 96-well plate, 5 μL of beads bearing 100 μM of **tetAcK-yne** were incubated with 25 mM Tris pH 8, 10 μM of **qSeDAPtri**, and varying concentrations of Ldt_Mt2_, respectively 0.1, 0.5, 1, and 2 μM of Ldt_Mt2_. The beads were thoroughly mixed by pipetting prior to incubation with shaking at room temperature for 1 h. Negative control wells included all components except the enzyme. The addition of 0.1% TFA stopped enzymatic catalysis after the incubation period. The beads were subsequently washed thrice by centrifugation at 4000 × *g* for 3 min with PBS containing 0.01% Tween-20. Bead fluorescence was assessed by an Attune NxT flow cytometer using the 488 nm laser and the 525/49 bandpass filter, as described previously.

### Ldt-Catalyzed Cross-Linking of **tetAck-yne** with **qSeDAPtri**

In a round-bottom, untreated 96-well plate, 5 μL of beads bearing 100 μM of **tetAcK-yne** were incubated with 25 mM Tris pH 8, 10 μM of **qSeDAPtri,** and 1 μM of Ldt_Mt2_. Negative control wells included all components except the enzyme. Inhibition of the enzyme was performed through treatment with 20 μM of freshly prepared meropenem. The beads were thoroughly mixed by pipetting prior to incubating them with shaking at room temperature for 1 h. The addition of 0.1% TFA stopped enzymatic catalysis after the incubation period. The beads were subsequently washed thrice by centrifugation at 4000 × *g* for 3 min with PBS containing 0.01% Tween-20. Bead fluorescence was assessed by an Attune NxT flow cytometer using the 561 nm laser and the 585/16 bandpass filter, as described previously.

### Bead-Based Cross-Linking of Peptide Substrates by *M. smegmatis* Ldts

In a round-bottom, untreated 96-well plate, 5 μL of beads bearing 100 μM of **tetAcK-yne** were incubated with 25 mM Tris pH 8, 10 μM of **qSeDAPtri**, and 1 μM of Ldt A-F. Negative control wells included all reagents except the enzyme. Inhibition of the enzyme was performed through treatment with 20 μM of freshly prepared meropenem. The wells were thoroughly mixed by pipetting prior to incubating them with shaking at room temperature for 1 h. The addition of 0.1% TFA stopped enzymatic catalysis after the incubation period. The beads were subsequently washed thrice by centrifugation at 4000 × *g* for 3 min with PBS containing 0.01% Tween-20 (PBST). Bead fluorescence was assessed by an Attune NxT flow cytometer using the 488 nMm laser and the 525/49 bandpass filter, as described previously.

### Bead-Based Cross-Linking of Acyl Acceptor Analogues with *Mtb* Ldt_Mt2_ and *M. smegmatis* Ldts

In a round-bottom, untreated 96-well plate, 5 μL of beads bearing 100 μM of **tetAcK-yne** were incubated with 25 mM Tris pH 8, 10 μM of **acyl acceptor**, and 1 μM of Ldt_Mt2_, LdtA-F. Negative control wells included all reagents except the enzyme. The wells were thoroughly mixed by pipetting prior to incubating them with shaking at room temperature for 1 h. The addition of 0.1% TFA stopped enzymatic catalysis after the incubation period. The beads were subsequently washed thrice by centrifugation at 4000 × *g* for 3 min with PBS containing 0.01% Tween-20 (PBST). Bead fluorescence was assessed by an Attune NxT flow cytometer using the 488 nm laser and the 525/49 bandpass filter as described previously.

### Z′ Analysis

In a round-bottom, untreated 96-well plate, 5 μL of beads bearing 100 μM of **tetAcK-yne** were incubated with 25 mM Tris pH 8, 10 μM of **qSeDAPtri**, and 1 μM of Ldt_Mt2_ (positive control, *n* = 12). Negative control wells (*n* = 12) included all reagents except the enzyme. The wells were thoroughly mixed by pipetting prior to incubating them with shaking at room temperature for 1 h. The addition of 0.1% TFA stopped enzymatic catalysis after the incubation period. The beads were subsequently washed thrice by centrifugation at 4000 × *g* for 3 min with PBS containing 0.01% Tween-20 (PBST). Bead fluorescence was assessed by an Attune NxT flow cytometer with the 488 nm laser and the 525/49 bandpass filter, as described previously. The Z′ score was calculated from replicate measurements of positive and negative control wells as one minus three times the sum of the standard deviations of the positive and negative controls divided by the absolute difference between their mean signals. (Z′ = 1 – [3­(σ_p_ + σ_n_)/|μ_p_ – μ_n_|])

### Antibiotic Inhibition of *M. smegmatis* Ldts and *Mtb* Ldt_Mt2_

In round-bottom untreated 96-well plates, purified Ldts (*M. smegmatis* LdtA,B,E,F and *Mtb* Ldt_Mt2_) at a concentration of 1 μM were incubated with 25 mM Tris pH 8 and varying concentrations (0, 0.01, 0.1, 0.5, 1, 5, 10, and 20 μM) of antibiotic for 20 min. The solutions were thoroughly mixed by pipetting prior to incubation. Then, 5 μL of **tetAcK-yne** beads and 10 μM of **qSeDAPtri** were added and allowed to shake for 1 h at room temperature. The addition of 0.1% TFA stopped enzymatic catalysis after the incubation period. The beads were subsequently washed thrice by centrifugation at 4000 × *g* for 3 min with PBS containing 0.01% Tween-20 (PBST). Bead fluorescence was assessed by an Attune NxT flow cytometer using the 488 nm laser and the 525/49 bandpass filter as described previously. The antibiotics tested included meropenem, faropenem, tebipenem, doripenem, biapenem, ceftriaxone, aztreonam, and ampicillin. Each antibiotic solution was freshly prepared in Milli-Q water as a 10 mg/mL stock solution immediately prior to each experiment and diluted to the desired concentrations. IC_50_ values were determined using GraphPad. Data points were plotted as the mean with standard deviation as the error bars. The values were normalized against the mean average of no-inhibitor controls and the mean average of no-enzyme controls. The dose–response analysis was performed by using the log­(inhibitor) vs. normalized response–variable slope model in GraphPad.

## Results and Discussion

### Assay Design for Probing Ldt Activity

Several pathogenic bacteria, including mycobacteria, incorporate the unusual amino acid *m*-DAP at the third position of the PG muropeptide (Figure 2a).^57,58^ The inherent symmetry of this building block presents a significant challenge for synthesis. As a result, protected *m*-DAP suitable for solid-phase peptide synthesis (SPPS) is not commercially available, and existing synthetic routes are lengthy and proceed with low overall yield.^59–64^ These difficulties in accessing *m*-DAP have greatly hampered the development of chemical probes designed to study *m*-DAP-containing structures and pathways. To address these challenges, we and others have developed stem peptide-mimicking chemical probes, including variants employing structural surrogates such as *meso*-cystine (*m*-CYT) and *meso*-selenolanthionine (*m*-SeLAN) in place of *m*-DAP (Figure 2a).^44,47,55,65–69^ These analogues are well-tolerated across multiple bacterial species, including mycobacteria, and have since enabled interrogation of Ldt activity within the mycobacterial cell wall in live cells.^55,65^

**Figure 2:**
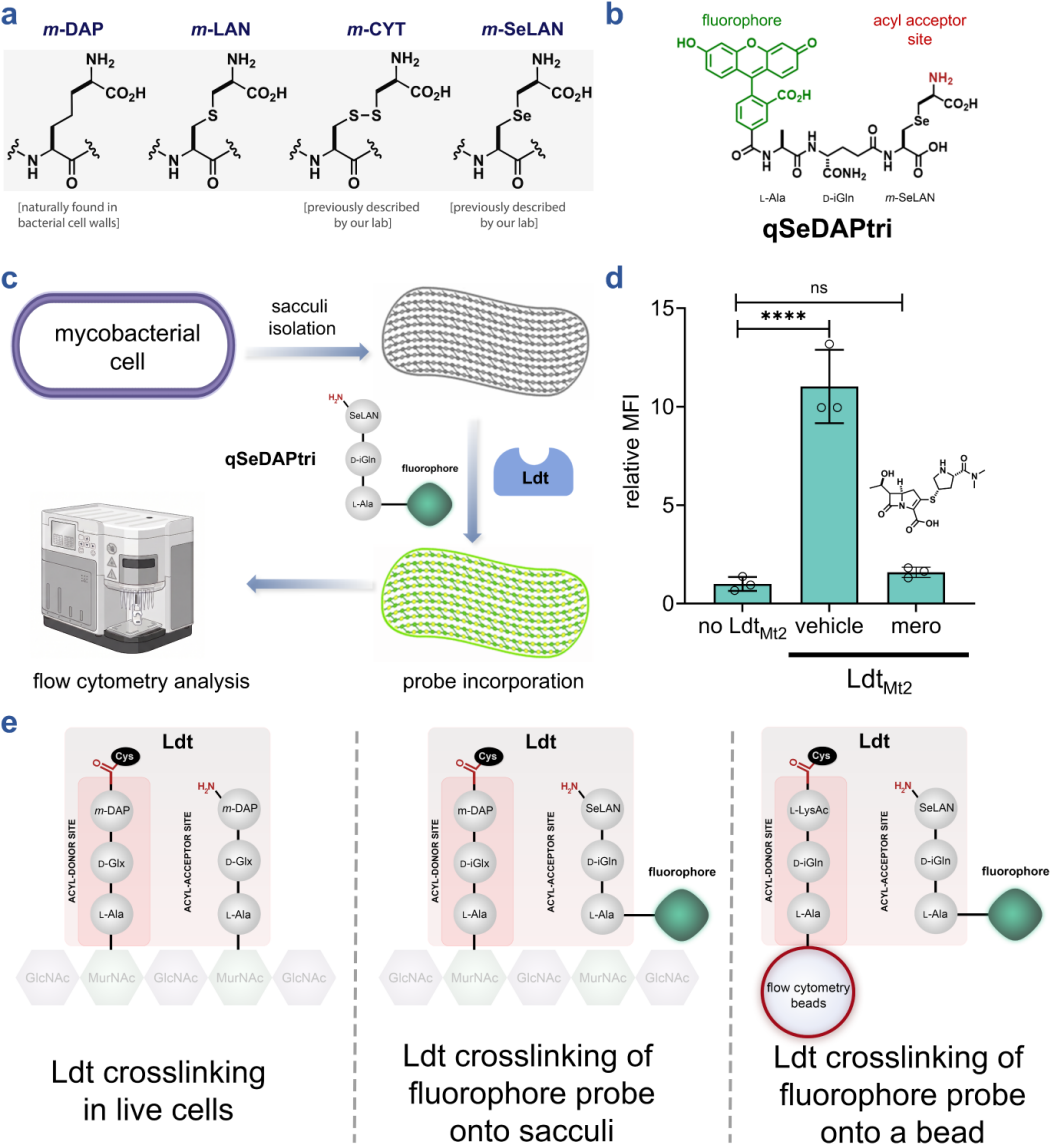
Assessment of Ldt-catalyzed cross-linking of fluorescent acyl acceptor onto *M. smegmatis* sacculi. (**a**) Schematic representation of *m*-DAP and the analogues incorporated within the PG of mycobacteria. (**b**) Schematic of the fluorescent tripeptide acyl acceptor **qSeDAPtri.** (**c**) PG sacculi is isolated from other cellular components of *M. smegmatis*. Once isolated, incubation of **qSeDAPtri** and the purified Ldt results in fluorescent sacculi that can be analyzed by flow cytometry. (**d**) Flow cytometry analysis of isolated PG sacculi incubated with 20 μM **qSeDAPtri** in the presence, and absence of 2 μM Ldt_Mt2_ for 2 h. Mean fluorescence intensity (MFI) represents the ratio of fluorescence relative to the control (no Ldt_Mt2_), quantified from 10000 events. Statistical significance was determined by one-way ANOVA (ns = not significant, **** *p* < 0.0001). The vehicle condition includes tetAcK-yne beads, qSeDAPtri, and Ldt_Mt2_ in the absence of inhibitor. (**e**) Illustrated overview depicting stem peptide processing within bacterial PG in vivo, incorporation of fluorescent stem peptide mimics into isolated PG sacculi, and probe cross-linking to a single acyl acceptor stem peptide mimic immobilized on an abiotic surface.

We leaned on these prior efforts to more faithfully mimic native Ldt substrates, including the synthesis of a fluorescent tripeptide acyl acceptor strand (**qSeDAPtri**, Figure 2b). Restricting the substrate to a tripeptide ensured that it functioned exclusively as an acyl acceptor, as it lacks the terminal d-Ala required to serve as an acyl donor during the cross-linking reaction. In addition, we included a fluorophore on the *N*-terminus of the probe, which is a site within muropeptide probes that we previously showed tolerates modification and can still be processed by Ldts.^55,70^ To test **qSeDAPtri** processing by Ldts, we initially reasoned that isolated sacculi could serve as the acyl donor chains (Figure 2c). Importantly, the use of sacculi, as a highly diverse and comprehensive set of acyl donors, can potentially offer unique insights into the breadth of substrates that can be operated on by Ldts. Incorporation of **qSeDAPtri** via Ldt-mediated cross-linking was expected to result in higher levels of sacculi-associated fluorescence; this step can readily be monitored by flow cytometry (SaccuFlow), a platform we recently disclosed to broadly enable higher-throughput detection of structural changes in isolated sacculi from various species of bacteria, including mycobacteria.^71,72^

As a proof of principle for sacculi-based analysis, we focused on Ldt_Mt2_, the best-characterized Ldt in *Mtb*, which contributes to cell wall integrity during growth and stress and represents an important target for antibacterial drug development.^13–15,73–77^ To benchmark our system, *M. smegmatis* sacculi were isolated using previously established protocols.^53,55,78–80^ Ldt_Mt2_ was subsequently incubated with sacculi and **qSeDAPtri**, and the resulting sacculi-associated fluorescence was monitored by flow cytometry. Our results showed that incubation of isolated mycobacterial sacculi with **qSeDAPtri** and Ldt_Mt2_ yielded a marked fluorescence increase over sacculi treated with the probe in the absence of the Ldt enzyme, confirming assay feasibility (Figure 2d). To confirm that fluorescence resulted from Ldt_Mt2_ enzymatic activity, we pretreated the enzyme with meropenem. Meropenem has been previously shown to block processing by Ldts by covalent trapping of the active site cysteine.^20,75,81^ In our platform, meropenem treatment reduced sacculi-associated fluorescence to near-background levels, demonstrating that this approach enables high-throughput discovery of novel Ldt inhibitors. In principle, this strategy can also be adapted to probe species- and strain-specific acyl donor preferences through sacculi isolation.

While sacculi provide several advantages for probing Ldt activity (e.g., accessibility, native acyl-strand composition, and facile isolation by standard laboratory techniques), the structural heterogeneity of acyl-donor substrates (present as muropeptides within the sacculus) limits the ability to conduct rigorous structure–activity relationship analyses of inhibitors and substrates (Figure 2e). To this end, muropeptides, although initially biosynthesized as canonical pentapeptides of uniform composition, subsequently undergo extensive structural modifications that include the removal of several amino acids (generating tetrameric, trimeric, dimeric, and denuded strands), amidation of D-*i*Glu residues, and incorporation of noncanonical amino acids in the fourth and fifth positions.^7,82^ Additionally, PG-associated molecules, including lipoteichoic acids and wall teichoic acids in Gram-positive bacteria, or the mycolyl-arabinogalactan layer in mycobacteria, must be removed to ensure enzyme access.^51,53,83–87^ Thus, isolating muropeptides reflective of mycobacterial PG diversity is not only time-consuming but also introduces batch-to-batch variability, ultimately limiting reproducibility and throughput.^53,85,88^ These limitations prompted us to develop an alternative strategy. In this iteration, a synthetic acyl donor peptide is immobilized onto polystyrene beads, and Ldt-mediated cross-linking of a fluorescent acyl acceptor is quantified by flow cytometry. Critically, both acyl donors and acyl acceptor strands are synthetic with a precisely defined structure. This approach recapitulates both catalytic steps: enzyme acylation by the bead-bound donor (with d-Ala release) and subsequent cross-link formation with the fluorescent acceptor, rendering beads fluorescent.

To implement this bead-based strategy, we synthesized an acyl donor peptide bearing an *N*-terminal propargyl group (**tetAcK-yne**, Figure 3a). The alkyl group was used to covalently link the synthetic acyl donor strand to azide-functionalized polystyrene beads via copper-catalyzed azide–alkyne cycloaddition (CuAAC). To ensure **tetAcK-yne** functioned exclusively as the acyl donor, we acetylated the lysine ε-amino group, thereby preventing it from acting as an acceptor.^55,70^ We first validated this chemical step by performing CuAAC with tetFAMK-yne, a peptide where fluorescein replaced the acetyl group on the lysine of tetAcK-yne, onto the azide-bearing beads and analyzing the fluorescence levels of the beads by flow cytometry (Figure S1). Upon the addition of CuAAC reagents, there was a marked increase in bead-associated fluorescence. After confirming successful bead functionalization via click chemistry, we conducted a series of experiments to optimize transpeptidation reactions using the bead-based reporter system (Figure 3b). As with the sacculi experiments, **qSeDAPtri** again served as the fluorescent acyl acceptor substrate for Ldt_Mt2_-mediated cross-link analysis. In the absence of the enzyme, bead fluorescence levels were low, suggesting minimal nonspecific binding of **qSeDAPtri** onto the bead support (Figure 3c). Similarly, the incubation of the beads with Ldt_Mt2_ but in the absence of **tetAcK-yne** resulted in similarly low levels of bead fluorescence, which indicates that the fluorescence must originate from cross-link formation with the acylated strand. **TetAcK-yne**-linked beads incubated with both the acyl acceptor strand and the enzyme resulted in a significant increase in bead-associated fluorescence. Upon the pretreatment of Ldt_Mt2_ with the β-lactam meropenem, bead fluorescence returned to background levels. Moreover, cross-link formation by Ldt_Mt2_ was performed with both substrates in solution and validated by high-resolution mass spectrometry (Figure S2). Together, these results confirmed that our platform specifically reports Ldt-catalyzed transpeptidation.

**Figure 3:**
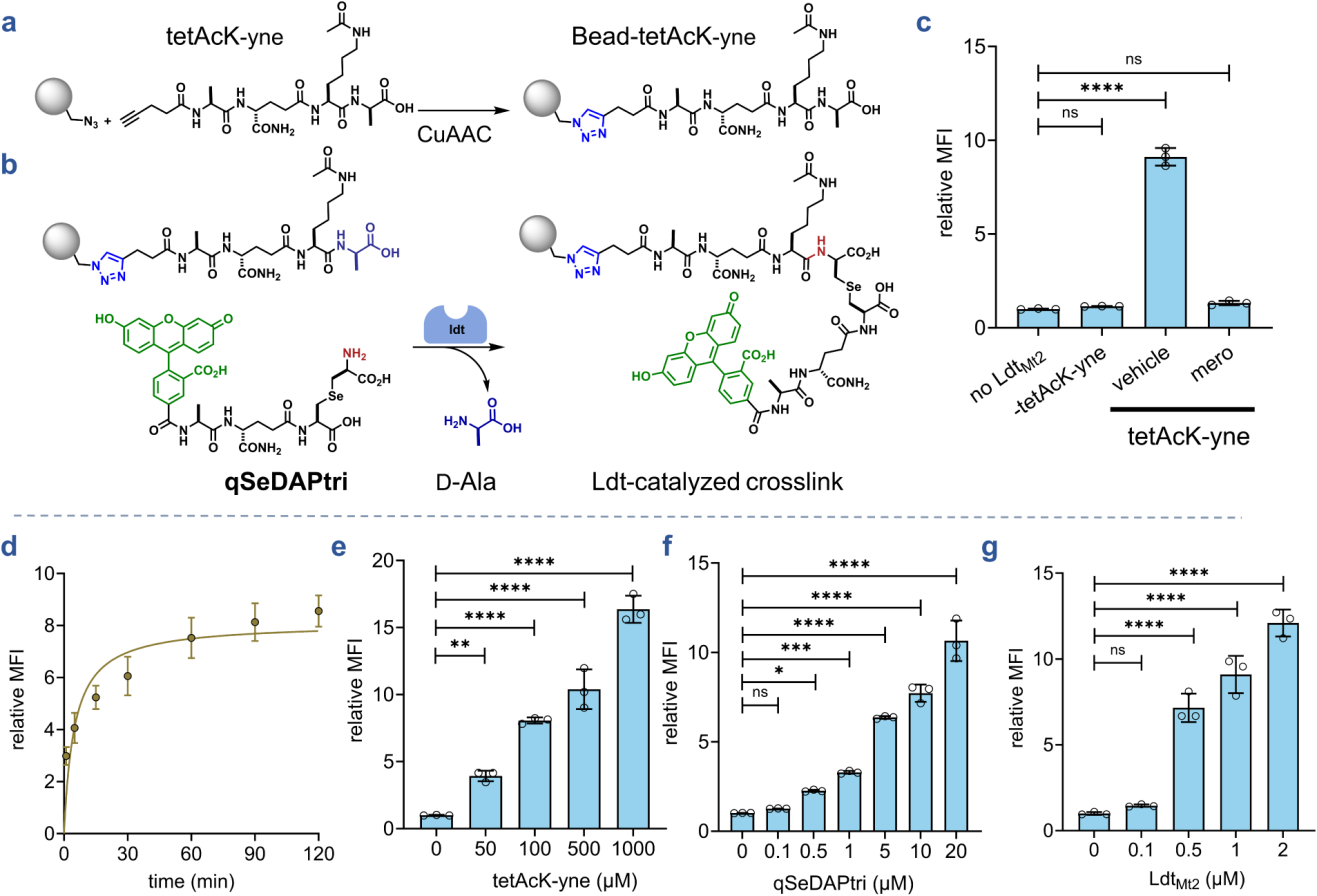
(**a–b**) Schematic representation of the l,d-transpeptidation assay. A fluorescent acyl-donor PG stem-peptide mimic (**tetAcK-yne**) was immobilized onto flow-cytometry–compatible polystyrene beads via copper-catalyzed azide–alkyne cycloaddition (CuAAC). Incubation of bead-bound **tetAcK-yne** with a fluorescent acyl-acceptor peptide (**qSeDAPtri**) and an Ldt results in enzyme-mediated cross-linking of the fluorescent peptide onto the bead surface, with the loss of the terminal d-alanine residue from **tetAcK-yne**. Fluorescence, reflecting Ldt activity, will be quantified by flow cytometry. (**c**) Flow cytometry analysis of beads bearing 100 μM **tetAcK-yne** incubated with 20 μM **qSeDAPtri** in the presence, absence, or inhibition of 2 μM Ldt_Mt2_ by meropenem for 2 h. (**d**) Time course of cross-linking. Beads bearing 100 μM **tetAcK-yne** were incubated with 20 μM **qSeDAPtri** and 2 μM Ldt_Mt2_ over 120 min intervals. The vehicle condition includes tetAcK-yne beads, qSeDAPtri, and Ldt_Mt2_ in the absence of inhibitor. (**e**) Concentration scan of acyl-donor loaded on beads. Beads bearing 50, 100, 500, or 1000 μM **tetAcK-yne** were incubated with 20 μM **qSeDAPtri** and 2 μM Ldt_Mt2_ for 1 h. (**f**) Concentration scan of acyl-acceptor substrate. Beads bearing 100 μM **tetAcK-yne** were incubated with 0.1, 0.5, 1, 5, 10, or 20 μM **qSeDAPtri** and 2 μM Ldt_Mt2_ for 1 h. (**g**) Enzyme-concentration scan. Beads bearing 100 μM **tetAcK-yne** were incubated with 5 μM **qSeDAPtri** and 0.1, 0.5, 1, or 2 μM Ldt_Mt2_ for 1 h. Mean fluorescence intensity (MFI) represents the ratio of fluorescence relative to the control (no Ldt_Mt2_), quantified from 10000 events. Statistical significance was determined by one-way ANOVA (ns = not significant, **** *p* < 0.0001).

### Systematic Optimization of Assay Parameters

Having established proof-of-concept, we systematically profiled reaction conditions to maximize assay sensitivity and the dynamic range. We first determined the optimal reaction time by monitoring Ldt_Mt2_ activity over time. Reactions were quenched with PBS containing trifluoroacetic acid, which protonates the catalytic cysteine to prevent further catalysis. For all assays, upon the completion of the assay period, beads were washed with Tween-20 containing buffer to remove noncovalently associated **qSeDAPtri**. Time-course analysis revealed the fluorescence plateaued after approximately 1 h (Figure 3d), which we adopted for subsequent experiments. We next probed how the **tetAcK-yne** concentration could modulate the dynamic range. Azide-functionalized beads underwent CuAAC with varying **tetAcK-yne** concentrations, followed by incubation with the Ldt enzyme and **qSeDAPtri**. Our data showed that 100 μM of **tetAcK-yne** yielded robust signal levels (Figure 3e), balancing substrate availability with background minimization. **qSeDAPtri** concentration was probed by incubating **tetAcK-yne** beads with Ldt_Mt2_ and varying acyl acceptor concentrations (Figure 3f). A clear concentration-dependent increase in fluorescence was observed with increasing levels of the acyl acceptor substrate. Finally, we tested various enzyme concentrations and found that there was apparent saturation at higher enzyme concentrations (Figure 3g). Combined, these results establish the feasibility of conjugating a synthetic, chemically defined acyl donor substrate to a flow cytometry-compatible bead and analyzing cross-linking with a synthetic, chemically defined acyl acceptor substrate.

To investigate the potential for multiplexing applications, we tested the flexibility of this mode of assay with other dyes. The fluorescein dye on the *N*-terminal was replaced with tetramethylrhodamine (**Tm_acceptor**) (Figure S3). Incubation of the TAMRA-labeled acyl acceptor strand with bead-bound **tetAcK-yne** and Ldt_Mt2_ led to a pronounced fluorescence increase over background. As seen with **qSeDAPtri**, inclusion of meropenem completely abolished the signal from the **Tm_acceptor** probe, confirming enzyme-dependent fluorescence generation. We next examined the effect of temperature, noting that Ldt_Mt2_ operates at human physiological temperature.^89^ Conducting reactions at 37 °C under optimized conditions produced robust, statistically significant signals comparable to room temperature results (Figure S4), confirming assay robustness under physiological conditions. Although an approximately 2-fold increase in MFI was observed at room temperature, this difference likely reflects temperature-dependent effects on the assay readout rather than intrinsic changes in Ldt activity. Consistent with prior reports,^32,35,36,38,39,56^ all subsequent assays were performed at room temperature.

### Expanding Detection Modalities: Antibody-Based Readouts

Having established the bead-based fluorescence assay, we next explored the possibility of using an orthogonal detection method to enhance versatility. Because fluorescein is a well-characterized antigen with high-affinity antibodies,^90,91^ we hypothesized that antifluorescein antibody could provide alternative detection of Ldt-mediated cross-linking. Beads were incubated with **qSeDAPtri**, and upon completion of the incubation time with Ldt, beads were treated with anti-FITC IgG, which was subsequently detected using an HRP-fused secondary antibody. Beads were arrayed in standard 96-well plates, and colorimetric signal development was monitored using a microplate reader (Figure S5). The assay produced significant absorbance above the background only in the presence of all components. Signal loss upon enzyme omission or meropenem inhibition confirmed the feasibility of this ELISA-like format. To eliminate the bead-transfer step, we adapted the assay to azide-functionalized microtiter plates. Azide surface density was confirmed by conjugating FAM-alkyne via CuAAC, followed by indirect ELISA detection using anti-FITC IgG and HRP-conjugated secondary antibody (Figure S6a,b). Having validated plate functionalization, we immobilized **tetAcK-yne** on well surfaces and performed Ldt-mediated transpeptidation reactions (Figure S7a). Detection with anti-FITC IgG followed by HRP-conjugated secondary antibody yielded robust colorimetric signal above background (Figure S7b). These results indicated platform compatibility with multiple detection strategies, fluorescent or enzyme-linked, thereby expanding the analytical flexibility and high-throughput screening potential.

### Profiling Transpeptidase Activity across *M. smegmatis* Ldt Paralogs

Mycobacterial Ldts are classified into six classes based on conserved sequences and structural motifs, with additional biochemical variations among paralogs.^16,56^ *M. smegmatis*, a common *Mtb* surrogate, encodes six Ldt paralogs (LdtA–F) compared to five in *Mtb* (Ldt_Mt1–5_), all of which are proposed to contribute to PG integrity.^16,17,56,92^ Previously, isolated muropeptides were used to assess the transpeptidase activity of all 5 *Mtb* Ldts in solution, showing 3→3 cross-linking activity for all but one of the paralogs, namely the class 3 Ldt, Ldt_Mt3_.^31^ Additionally, genetic deletions of *M. smegmatis* Ldt paralogs reveal their ability to incorporate fluorescent d-amino acids within the PG or to determine the predominant Ldt within *M. smegmatis*.^16,92^ Beyond genetic manipulations of Ldt paralogs in *M. smegmatis*, LC/MS has been used to demonstrate differential acylation of all six paralogs by various β-lactams.^56^ As such, we leveraged our optimized bead-based platform to systematically profile the transpeptidase activity of these paralogs in vitro. All six *M. smegmatis* Ldts were purified and assessed under optimized conditions. LdtA, LdtB, LdtE, and LdtF generated significant fluorescence signals above background (Figure 4a, b, e, f). Upon pretreatment of the enzymes with meropenem, bead fluorescence returned to background levels. These results confirmed the Ldt-catalyzed activity of these enzymes for the substrates presented here. Notably, this represents, to our knowledge, the first demonstration of catalytic activity for a class 6 mycobacterial Ldt (LdtF) using purified peptidyl substrates, establishing our platform’s utility for characterizing previously uncharacterized enzymes.

**Figure 4:**
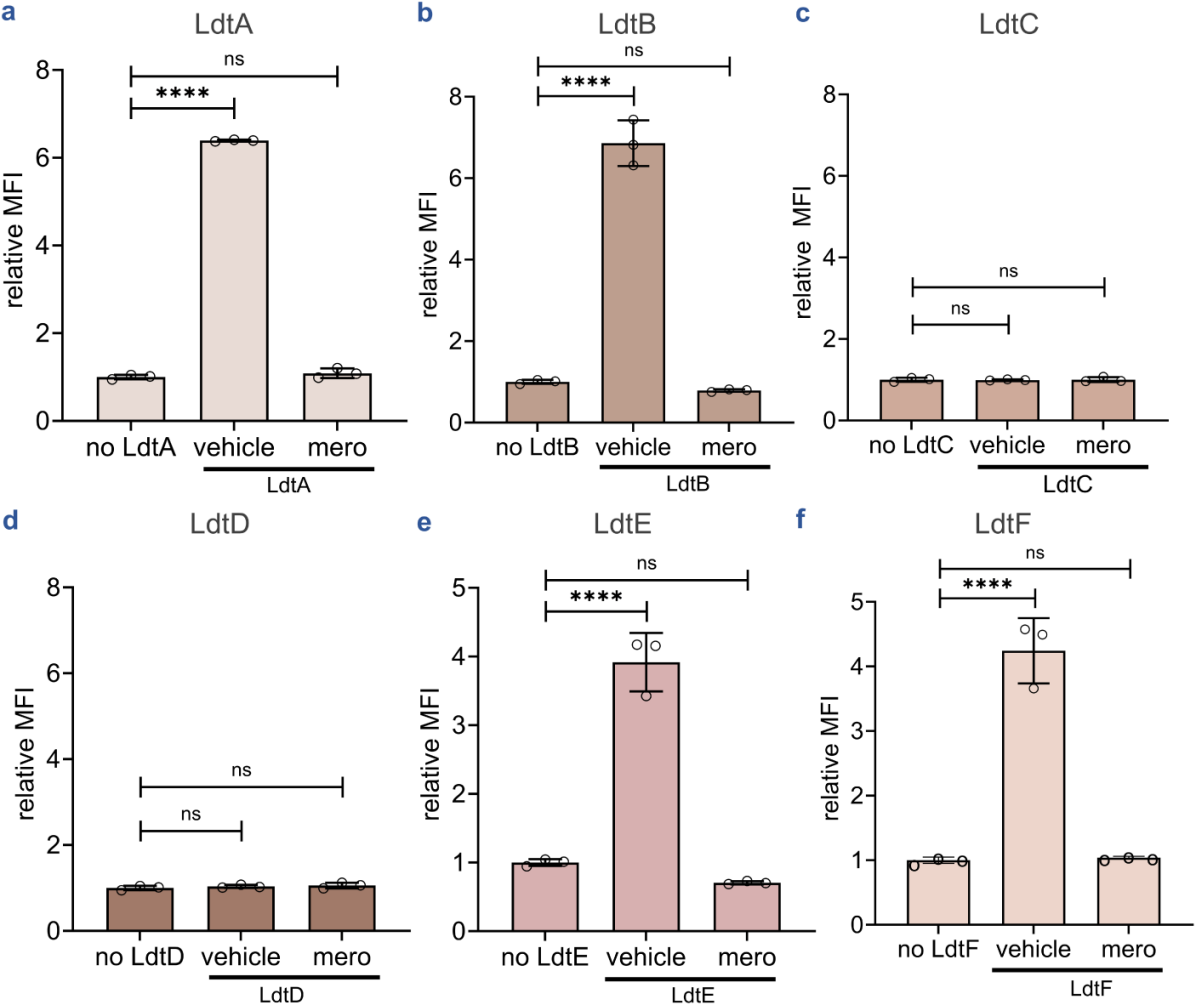
Assessment of transpeptidase activity of *M. smegmatis* Ldt paralogs. Flow cytometry analysis of beads bearing **tetAcK-yne** incubated with **qSeDAPtri** in the presence or absence of LdtA-F for 1 h (**a**–**f**). Mean fluorescence intensity (MFI) represents the ratio of fluorescence relative to the control (no Ldt), quantified from 10000 events. Statistical significance was determined by one-way ANOVA (ns = not significant, **** *p* < 0.0001). The vehicle condition includes tetAcK-yne beads, qSeDAPtri, and Ldt in the absence of inhibitor.

Interestingly, no significant signal above background was detected for class 3 enzyme LdtD or class 5 enzyme LdtC (Figure 4c,d). To our knowledge, there are no reports describing 3→3 cross-linking activity of class 3 Ldts. Conversely, the class 5 Ldt in *Mtb* (Ldt_Mt5_) has been previously reported to form 3→3 cross-linking in vitro, and LdtC can function as the sole 3→3 cross-linking enzyme in *M. smegmatis*.^17, 31^ Circular dichroism experiments were performed to rule out the possibility that LdtC inactivity was the result of improperly folded protein. Data indicate both LdtC and Ldt_Mt5_ have comparable CD spectra and indicate the protein is folded (Figure S8). Beyond 3→3 PG cross-linking, other Ldts have been found in different species of bacteria to have other roles, including anchoring and cleaving of membrane proteins to the PG^93–98^ unipolar cellular elongation,^96,99^ or unusual 1→3 PG cross-link^100,101^ In *E. coli*, LdtA (ErfK), LdtB (YbiS), and LdtC (YcfS) primarily catalyze the covalent attachment of Braun lipoprotein (Lpp) to PG, reinforcing the outer membrane.^93^ The class 3 Ldt enzyme (*M. smegmatis* LdtD) utilized here may have a function other than the canonical 3→3 cross-linking being monitored via the platform described here. Moreover, LdtD was shown to be acylated by (carba) penems.^56^ Conversely, the lack of detectable activity by the *M. smegmatis* Class 5 LdtC suggests it cannot efficiently process **qSeDAPtri**, even though its *Mtb* homologue, Ldt_Mt5_, forms 3→3 cross-links *in vitro*.^31^ We hypothesize that this reflects stringent substrate specificity, potentially requiring authentic *m*-DAP-containing acceptors rather than *m*-SeLAN surrogates. Future optimization incorporating *m*-DAP-based probes may elucidate substrate preferences for LdtC (and potentially LdtD) and expand the applicability of the platform for this Ldt class.

### Acyl Acceptor Profiling Reveals Chemical Modifications Tolerated by Ldts

Having established the bead-based assay, we next assessed the breadth of peptide substrates tolerated as acyl acceptors. We have previously shown that tripeptides bearing various residues at the third position function exclusively as acyl acceptors in live bacteria, as the absence of the d-alanyl residue prevents recognition by Ldts to function as a donor stem.^55,65^ In contrast, tetrapeptides containing a lysine residue at the third position are well-tolerated by Ldts and are efficiently incorporated within the PG of live bacteria as either acyl donors or acceptors.^52,70,79,80^ Accordingly, our platform, employing a single acyl donor (tetAcK-yne, Figure 3a) and a single acyl acceptor, is well-suited to interrogate how chemical modifications influence Ldt recognition and processing of fluorescent acyl acceptors. While our prior cellular studies provided critical validation of probe uptake and incorporation, these live-cell analyses inherently expose PG analogues to the full suite of active transpeptidases and a vast diversity of acyl-complementary ends (Figure 2e). This environmental complexity makes it nearly impossible to systematically dissect how specific structural motifs drive the enzymatic function.

To rigorously map the determinants of substrate recognition in a controlled environment, we synthesized a library of eight additional stem peptide analogues that reflect the natural heterogeneity of mycobacterial PG (Figure 5a). These probes were designed to systematically evaluate modifications critical to cell wall architecture, including the amidation status of the *iso*-d-glutamic acid and the identity of the third-position diamino acid (contrasting *m*-DAP with lysine, a hallmark distinction between mycobacterial and other bacterial chemotypes). We also investigated the impact of peptide chain length on acyl-acceptor competence by testing trimeric, tetrameric, and pentameric variants. As before, we initiated our evaluation with Ldt_Mt2_ to establish a baseline for catalytic tolerance. Evaluation of trimeric substrate revealed that Ldt_Mt2_ tolerated only those harboring an *m*-DAP mimetic at the third position, **qSeDAPtri** and **qCYTtri**, consistent with prior observations in live bacteria.^55,65^ Importantly, the lack of fluorescent signal from **qSeLYStri** underscores the requirement of a side-chain carboxylate in trimeric substrates to enable a productive engagement within the active site of this enzyme, as we previously demonstrated in live mycobacteria.^55^

**Figure 5:**
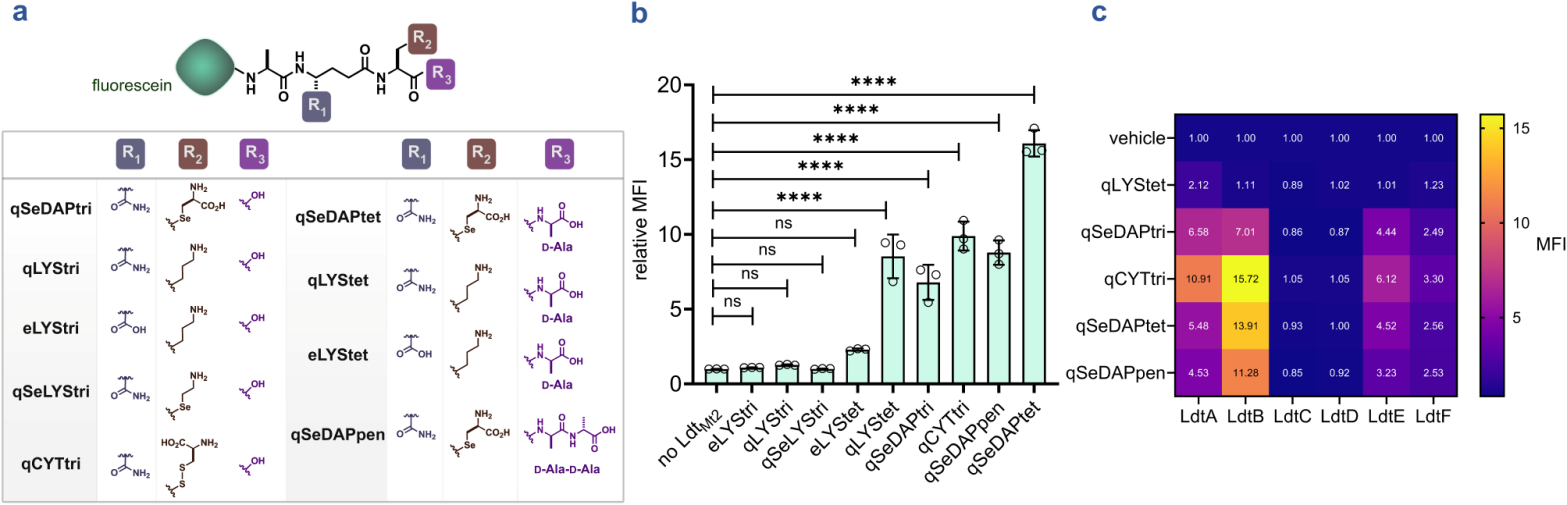
Systematic evaluation of acyl-acceptor tolerance across Ldt enzymes. (**a**) Schematic of acyl acceptor stem peptide analogs used to probe substrate tolerance in *Mtb* Ldt_Mt2_ and across *M. smegmatis* Ldt paralogs. (**b**) Assessment of structural features within acyl acceptors tolerated by Ldt_Mt2_. Fluorescent PG stem-peptide analogs with defined compositional differences were evaluated for processing using bead-bearing **tetAcK-yne** and Ldt_Mt2_. Mean fluorescence intensity (MFI) represents the ratio of fluorescence relative to the control (no Ldt), quantified from 10000 events. Statistical significance was determined by one-way ANOVA (ns = not significant, **** *p* < 0.0001). (**c**) Heatmap summarizing the processing of acyl acceptors by *M. smegmatis* Ldt paralogs, as determined using the bead-based assay under optimized conditions. Data represent the mean values from three independent replicates (*n* = 3). *p*-values were determined by one-way ANOVA (ns = not significant, **** *p* < 0.0001).

We have previously established that fluorescent and bioorthogonal tetrapeptides bearing a third-position lysine are incorporated into the PG of mycobacteria.^70, 79, 80^ Consistent with these findings, assessment of tetrameric acyl acceptors in our assay revealed that Ldt_Mt2_ tolerated all tetrameric substrates except **eLYStet**, which we attribute to the lack of amidation of the *iso*-d-glutamic acid at the second position. Together, these results underscore the importance of *iso*-d-glutamic acid amidation in Ldt processing and highlight the critical role of the terminal d-alanine in substrate recognition and enzymatic catalysis (Figure 5b).^47–49,70^ Finally, we observed that **qSeDAPpen** was also tolerated by Ldt_Mt2_. We hypothesize that this tolerance arises from the presence of the *m*-DAP mimetic at the third position, despite the substrate being terminated by d,d-stereocenters that preclude Ldt engagement and its function as a donor stem (Figure 5b).^70^ While the probes presented here incorporate nonamidated *m*-DAP mimetics, we note that the free carboxyl of *m*-DAP is sometimes amidated in mycobacteria and may influence Ldt activity and cross-linking.^82^ Future studies incorporating amidated *m*-DAP mimetic probes could help elucidate its impact.

Having established the substrate scope of Ldt_Mt2_, we next evaluated the tolerance of these analogues across *M. smegmatis* Ldt paralogs. Peptides bearing a third-position *m*-DAP mimetic were well tolerated by LdtA, LdtB, LdtE, and LdtF (Figures 5c and S9). Only LdtA tolerated the tetrameric substrate, harboring a third-position lysine (Figures 5c, S9). In contrast, LdtC and LdtD failed to process any of the tested substrates. These results suggest more stringent substrate requirements for cross-linking and support their proposed roles in processes distinct from canonical PG peptide cross-linking. Collectively, these results demonstrate the utility of our platform to systematically interrogate structural modification tolerance across the Ldt paralogs.

### High-Throughput β-Lactam Profiling Validates Screening Potential

Having shown that our assay reports on Ldt-mediated cross-linking across multiple enzyme paralogs, we applied it to profile β-lactam antibiotic inhibition. To assess suitability for high-throughput screening, we first evaluated assay quality in 96-well format. **TetAcK-yne** beads were incubated with **qSeDAPtri** in the presence or absence of Ldt_Mt2_ to establish positive and negative controls. We calculated a Z′ score of 0.697 (Figure 6a, b), well above the 0.5 threshold, indicating adequate signal separation and reliability for screening applications. We then evaluated a representative β-lactam subset, including (carba)­penems, ampicillin, monobactams, and cephalosporins (Figure 6c and **Figure** S10). Each enzyme was preincubated with increasing antibiotic concentrations before assessing transpeptidase activity. For *M. smegmatis* Ldts and Ldt_Mt2_, we observed dose-dependent inhibition by meropenem, faropenem, doripenem, biapenem, and tebipenem, whereas ceftriaxone, aztreonam, and ampicillin exhibited minimal inhibition at tested concentrations (≤20 μM) (Figure 6d, Figure 6e, and Figure S10). Half-maximal inhibitory concentrations (IC_50_) for each enzyme-antibiotic pair were calculated and summarized (Table S1). Visualization of pIC_50_ values (negative log IC_50_) revealed distinct inhibition profiles across the β-lactam classes tested (Figure 6f). These findings align with previous reports that Ldts belonging to classes 1, 2, and 4 are preferentially inhibited by the carbapenem tebipenem and the penem faropenem, and that meropenem is the most potent LdtF (class 6) inhibitor, validating the accuracy of the platform.^19,33,35,36,56^ Finally, further demonstrating our platform’s reliability is the observed minimal activity of the penam ampicillin, the monobactam aztreonam, and the cephalosporin ceftriaxone within the tested concentration range, as these are known to be poor Ldt inhibitors.^35,36^

**Figure 6:**
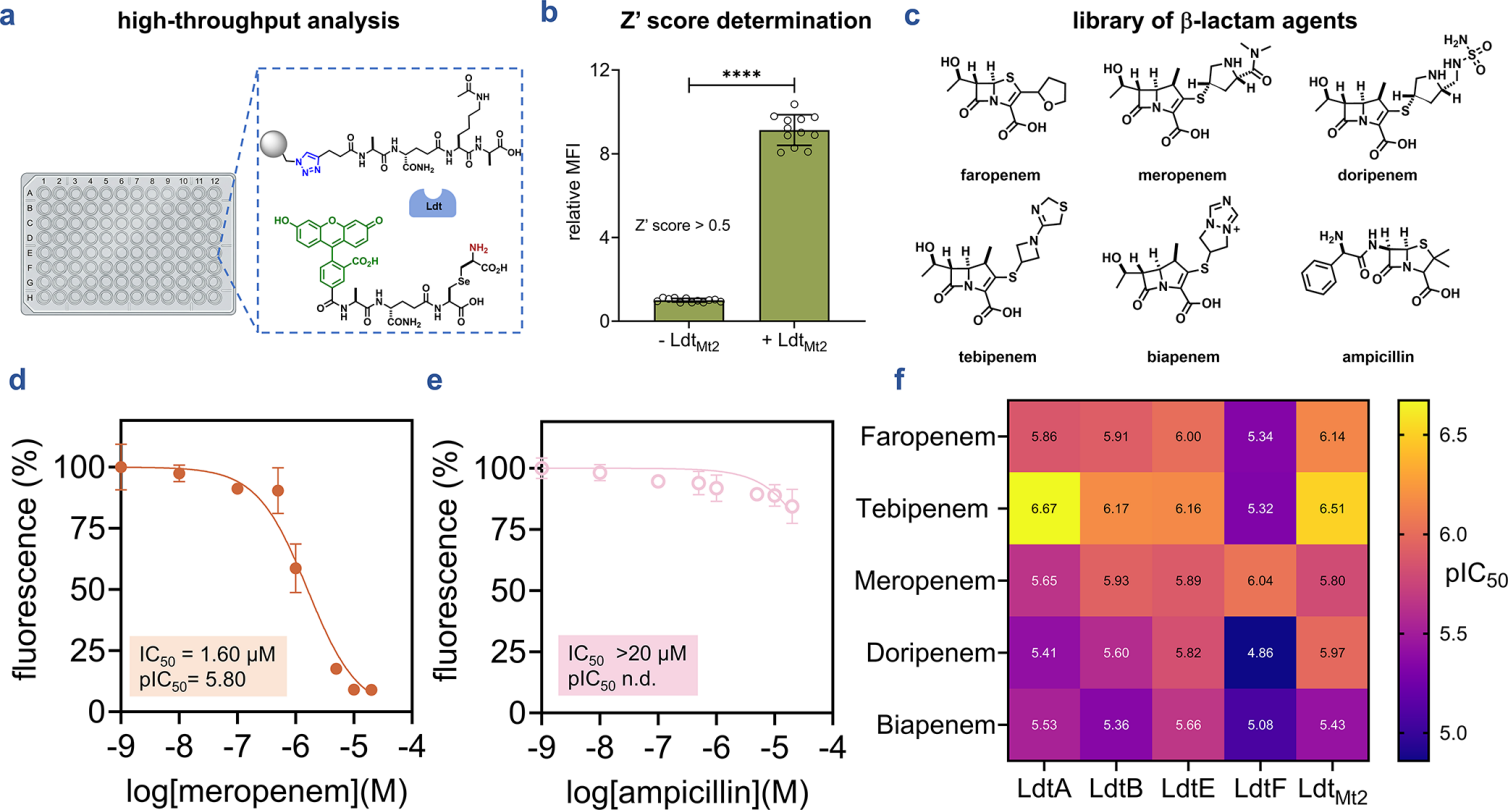
High-throughput bead-based profiling of antibiotic activity against l,d-transpeptidases. (**a**) Application of the bead-based assay platform for high-throughput screening. (**b**) Assessment of the separation between the positive (+ Ldt_Mt2_) and the negative control (− Ldt_Mt2_) for Z′ score determination. The calculated Z′ score was 0.697, above the 0.5 threshold, indicating reliable signal separation for screening applications. (**c**) Chemical structure of antibiotics spanning multiple β-lactams subclasses including (carba)­penem and penam. (**d**) Representative IC_50_ curve illustrating concentration-dependent inhibition of Ldt_Mt2_ by meropenem using the bead-based assay. (**e**) Lack of inhibitory activity of ampicillin against Ldt_Mt2_ under the same assay conditions. (**f**) Heatmap of pIC_50_ values summarizing inhibition of *M. smegmatis* Ldt paralogs and *Mtb* Ldt_Mt2_ by (carba)­penems, determined using the bead-based assay under optimized conditions. Data points represent the mean values of three replicates (*n* = 3).

## Conclusion

We have developed a versatile, high-throughput platform for investigating l,d-transpeptidase enzyme activity and inhibition. The system employs a bead-based format, where an acyl donor peptide substrate is immobilized on polystyrene beads, and enzyme-catalyzed incorporation of a fluorescent acyl acceptor tripeptide is quantitatively monitored by flow cytometry. This approach is readily adaptable to alternative detection modalities, including indirect ELISA configurations utilizing antibody-based readouts or immobilization of an acyl donor on microtiter plate surfaces with antibody-mediated chemiluminescent detection. This flexibility enhances the throughput and scalability for diverse screening applications.

Using this platform, we systematically profiled β-lactam antibiotic inhibition across multiple Ldt paralogs, including the primary transpeptidase of *Mtb,* Ldt_Mt2_. Results confirmed that (carba)­penems exhibit potent, concentration-dependent inhibition, thereby validating platform accuracy. Beyond inhibitor profiling, the system enabled evaluation of the structure–activity relationships of acyl-acceptor peptides across Ldt paralogs, and characterization of transpeptidase activity across *M. smegmatis* Ldt paralogs, revealing, catalytic activity of the class 6 enzyme LdtF with purified substrates. Conversely, lack of detectable activity for LdtC suggests stringent substrate specificity, highlighting opportunities for future probe optimization.

This platform overcomes critical limitations of existing Ldt activity assays, eliminating the dependence on laborious sacculi preparation while maintaining mechanistic fidelity to the native two-step transpeptidation reaction. The demonstrated compatibility with multiple detection modalities, robust performance under physiological conditions, and validated screening metrics establish this as a practical tool for accelerating next-generation therapeutic development. As antimicrobial resistance continues to escalate, particularly in mycobacterial infections, such tools are essential for discovering novel scaffolds with therapeutic potential against *Mtb* and related pathogens.

### Ldt UniProt Accession ID

ProteinOrganismUniProt IDLdt_Mt2_

*Mtb* H37Rv
I6Y9J2
LdtA
*M. smegmatis* mc^2^155
A0QY44
LdtB
*M. smegmatis* mc^2^155
A0R1G6
LdtC
*M. smegmatis* mc^2^155
A0QQZ4
LdtD (formerly LdtG)
*M.
smegmatis* mc^2^155
A0QQ95
LdtE
*M. smegmatis* mc^2^155
A0QP10
LdtF
*M. smegmatis* mc^2^155
A0QS24


## Statistical Analysis

Unless otherwise specified, statistical analysis was conducted by using GraphPad Prism 9.5. Experiments were conducted in triplicate, and significant experiments were repeated at least twice. One-way ANOVA was used to calculate statistical significance. Error bars represent the standard deviation.

## Supplementary Material



## Notes

Additional figures illustrating experimental data, complete synthetic routes for peptide and small molecule preparation, full characterization data (HRMS, HPLC) for all synthesized compounds, and supplementary references cited in the methodology and discussion (PDF)
